# Nemaline myopathy caused by*TNNT1* mutations in a Dutch pedigree

**DOI:** 10.1002/mgg3.52

**Published:** 2013-12-12

**Authors:** W Ludo van der Pol, Jolien F Leijenaar, Wim G M Spliet, Selma W Lavrijsen, Nicolaas J G Jansen, Kees P J Braun, Marcel Mulder, Brigitte Timmers-Raaijmakers, Kimberly Ratsma, Dennis Dooijes, Mieke M van Haelst

**Affiliations:** 1 Rudolf Magnus Institute of Neuroscience, Department of Neurology and Neurosurgery, University Medical Center Utrecht3584 EA, Utrecht, The Netherlands; 2 Department of Medical Genetics, University Medical Center Utrecht3584 EA, Utrecht, The Netherlands; 3 Department of Pathology, University Medical Center Utrecht3584 EA, Utrecht, The Netherlands; 4 Department of Pediatrics, Pediatric Intensive Care, Wilhelmina Children's Hospital, University Medical Center Utrecht3584 EA, Utrecht, The Netherlands

**Keywords:** Nemaline myopathy, *TNNT1*, troponin T1

## Abstract

Nemaline myopathy (NM) is genetically heterogeneous disorder characterized by early onset muscular weakness and sarcoplasmatic or intranuclear inclusions of rod-shaped Z-disk material in muscle fibers. Thus far, mutations in seven genes have been identified as cause of NM. Only one single*TNNT1* nonsense mutation has been previously described that causes autosomal recessive NM in the old order Amish with a very specific clinical phenotype including rapidly progressive contractures. Here, we report a patient who is compound heterozygous for a c.309+1G>A mutation and an exon 14 deletion in the*TNNT1* gene. This report confirms the specific clinical phenotype of*TNNT1* NM and documents two new*TNNT1* mutations outside the old order Amish.

## Introduction

Nemaline myopathy (NM [ MIM 256030, 161800] ) is a congenital myopathy characterized by early onset muscular weakness and rod-like inclusions of Z-disk material in muscle fibers. Typical cases have a congenital onset with hypotonia, predominantly proximal weakness of the extremities and respiratory insufficiency, often accompanied by facial weakness with sparing of the extraocular muscles (Wallgren-Pettersson et al. 2011; Nance et al. 2012). Mutations in seven genes have been described to cause NM; alpha-tropomyosin (*TPM3*), nebulin (*NEB*), alpha-actin (*ACTA1*), beta-tropomyosin (*TPM2*), Kelch-repeat, and BTB/POZ domains containing protein 13 (*KBTBD13*), cofilin-2 (*CFL2*), and troponin T1 (*TNNT1*) (Kiphuth et al. 2010; Wallgren-Pettersson et al. 2011; Nance et al. 2012). With the exception of*TNNT1*, mutations in these genes have a global distribution. Homozygosity for a single nonsense*TNNT1* NM mutation was identified in the old order Amish and is associated with a very specific clinical phenotype (Johnston et al. 2000). Amish NM (ANM) has a relatively high incidence among Amish and is, unlike other NM, characterized by early onset but subsiding tremors of the jaw and lower limbs, progressive contractures of the shoulders and hips, and a pectus carinatum. Respiratory insufficiency characteristically starts in the second year of life (Johnston et al. 2000). Here, we report the first patients outside the old order Amish community with clinical features strongly reminiscent of ANM.

The proband is the first child of healthy nonconsanguineous Caucasian parents. He was born after an uneventful pregnancy of 41 weeks with a birthweight of 3524 g. At the age 2 months, he presented with generalized stiffness, rigid spine, and reduced cervical rotation range. Passive movements of the shoulders, hips, and knee joints were severely limited and precluded assessment of muscle strength. Muscles of extremities felt atrophic, hand movements were normal. Reflexes could not be elicited and there were no tremors. He had a marked kyphosis, a rigid spine and thorax, and marked pectus carinatum, but no other dysmorphic features. Disease course was uneventful until he was admitted at the age of 2 years with pneumonia and respiratory insufficiency. There had been serious feeding difficulties in the weeks before admission. Chest X-rays showed infiltrative changes in the right lung and herniation of the stomach and intestines in the thoracic cavity. Treatment consisted of tracheostomy, chronic ventilation, and surgical correction of his diaphragmatic hernia followed by gastrostomy. He was discharged with chronic home mechanical ventilation. The family history reported two paternal cousins (their mother is the sister of the proband's father), a boy and a girl from nonconsanguineous parents, who died aged 3 and 5 years, respectively, due to respiratory insufficiency. They had a history of severe hypotonia and muscle weakness. Contractures of elbows and knees were reported in the boy and severe pectus carinatum with kyphosis and contractures of elbows and ankles in the girl. They had a postmortem diagnosis of NM after revision of the muscle biopsies.

**Figure 1:**
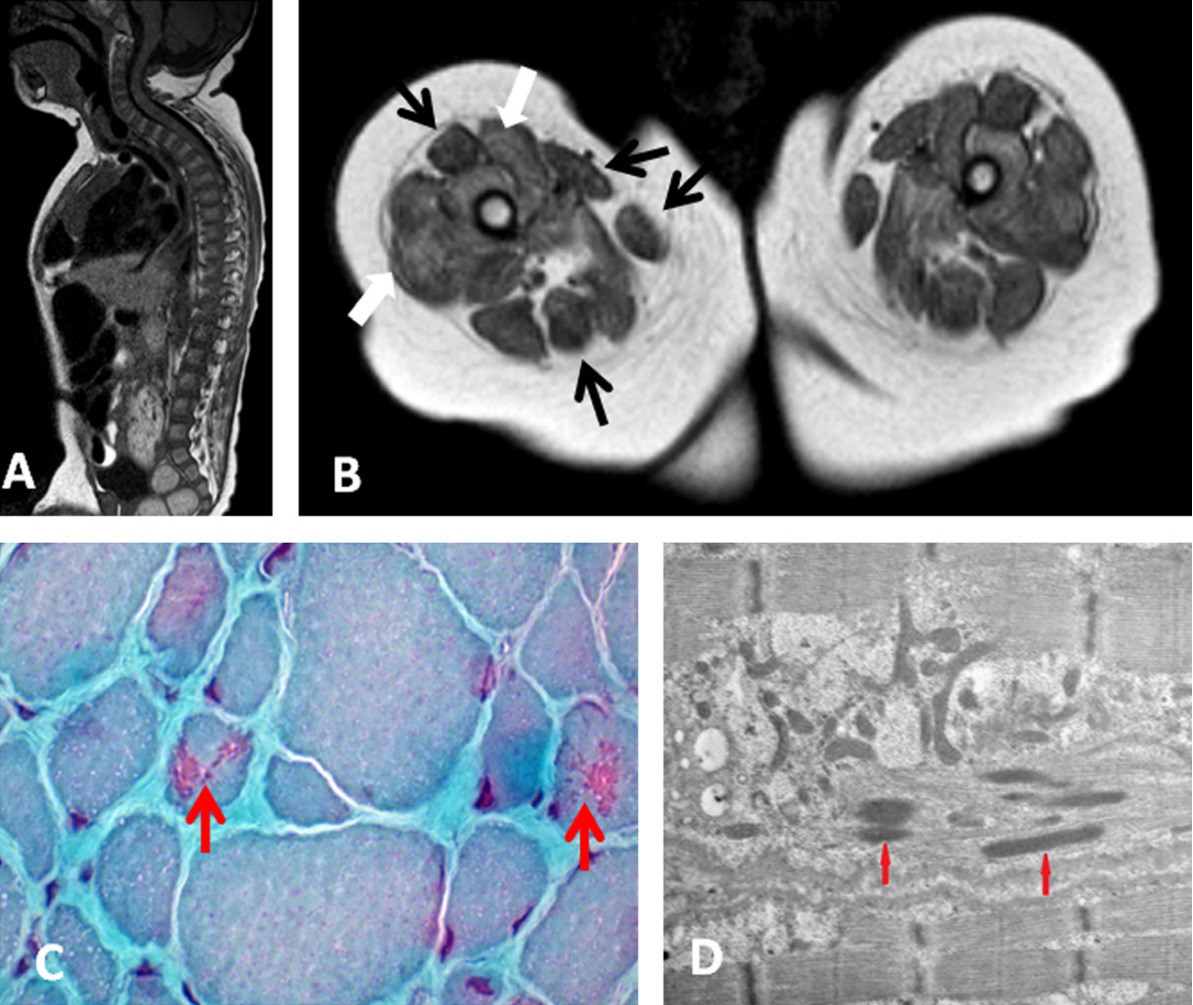
Magnetic resonance imaging (MRI) and muscle biopsy results. MRI showed marked kyphosis (A), and diffuse fatty infiltration of the anterior compartment of the upper leg (white arrows) with relative sparing of the rectus femoris, gracilis, and sartorius and posterior compartment (black arrows) (B). Gomori trichrome staining (C) showed variation in muscle fiber size and the presence of nemaline rods (arrows). Electron microscopy ultrastructural analysis (D) also showed nemaline rods (arrows).

Magnetic resonance imaging of the muscles and spine of the proband showed marked kyphosis (Fig. 1A) and increased T1 signal of the solei and glutei in the lower leg, adductor muscles, and vasti of the upper leg, with relative sparing of the rectus femoris, sartorius, gracilis, and hamstrings (Fig. 1B). Muscle biopsy from the lateral vastus showed marked variation in fiber size with smaller type 1 than type 2 fibers and nemaline rods in the Gomori stain and electron microscopy images (Fig. 1C and D).

**Figure 2:**
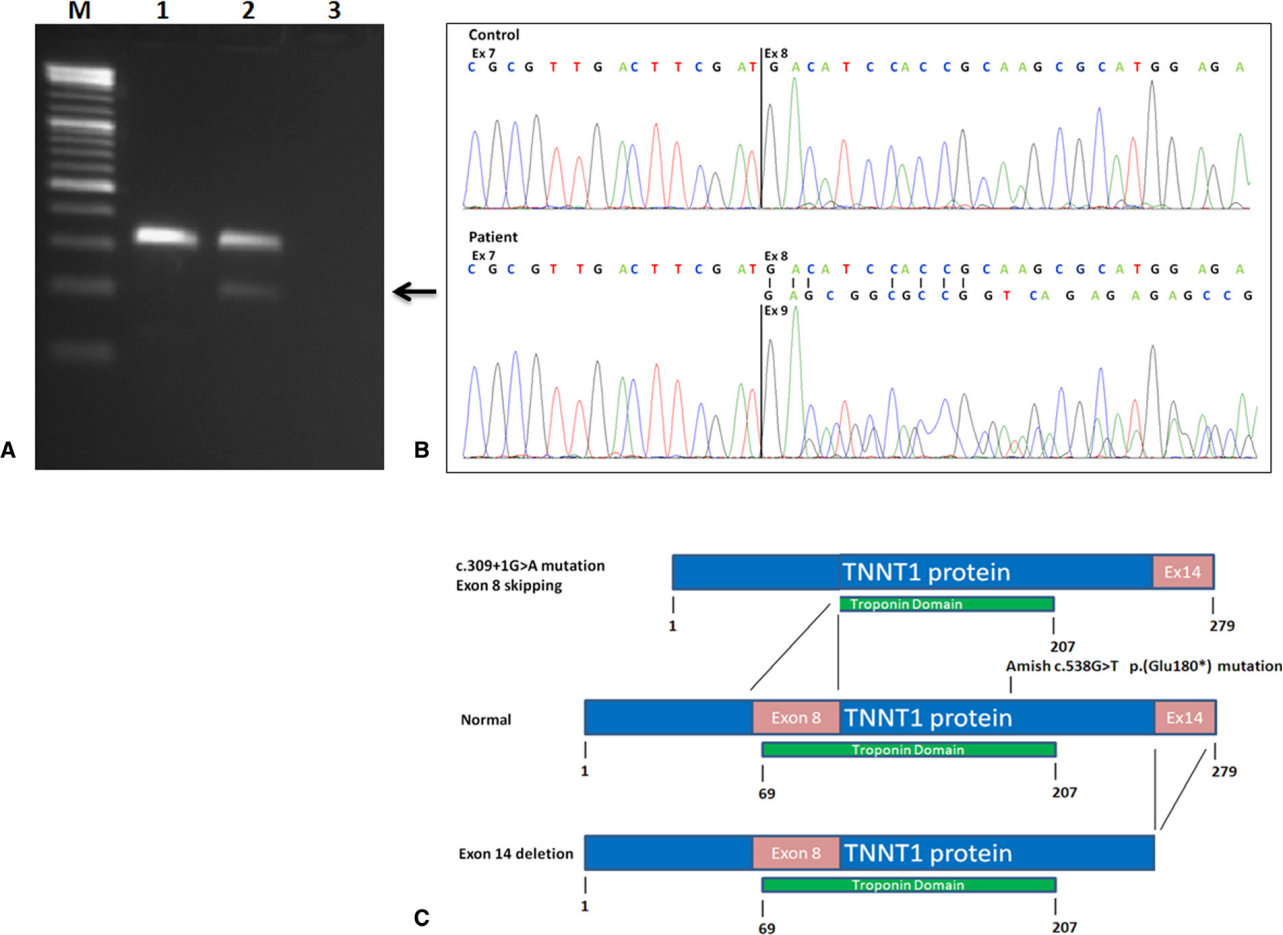
c.309+1G>A mutation leads to TNNT1 mRNA exon 8 skipping. (A) Agarose gel showing exon 7 to exon 10 RT-PCR products of control individual (lane 1), heterozygous father of index patient (lane 2), and water blank (lane 3). Lane M: 100 bp size ladder. Arrow indicating RT-PCR product from mutated allele with deletion of exon 8 (116 bp) from mRNA. (B) Control: chromatogram of RT-PCR fragments from Lane 1 (A). Patient: chromatogram from RT-PCR fragments from Lane 2 (A). RT-PCR analysis of heterozygous father (“Patient” sample, Lane 2) shows mix of exon 7–exon 8 mRNA fragments from the normal allele and exon 7–exon 9 fragments (arrow) showing exon 8 skipping from the mutated allele. (C) Schematic presentation of inferred effects on TNNT1 protein composition of the c.309G>A and exon 14 deletion mutations described in this study. Both mutations lead to truncated TNNT1 protein (or, alternatively to absense of mutated protein through NMD). For comparison, the position of the original Amish c.538G>T p.(Glu180*) mutation is shown.

Molecular analysis revealed no abnormalities in*ACTA1*,*CFL2*,*TPM2*,*TPM3*, and*KBTBD13*. A new heterozygous pathogenic mutation was identified in intron 8 of the*TNNT1* gene (c.309+1G>A). This mutation was inherited from the father. Maternal*TNNT1* sequence analysis did not reveal a pathogenic mutation. Molecular analysis of the DNA of the two deceased paternal cousins showed homozygosity for the pathogenic c.309+1G>A mutation confirming a postmortem diagnosis of*TNNT1* NM. The c.309+1G>A mutation is located at the invariant splice donor site of exon 8, predicting to result in*TNNT1* mRNA exon 8 skipping. To test this, we examined*TNNT1* expression in the father of the proband. Total RNA was isolated from paternal fresh blood samples using the PAX protocol (Qiagen Benelux, Venlo, the Netherlands) and subjected to reverse transcriptase-PCR. The obtained cDNA products were PCR amplified using primers specific for*TNNT1* exons 7 and 10. PCR fragments were size separated using 2% agarose gel electrophoresis. In addition, the PCR fragments were used for direct sequence analysis. Primers and PCR details are available upon request. In addition to a normally sized PCR fragment including exons 7-8-9-10, the father of the proband showed a 116 nucleotides smaller PCR fragment (Fig. 2A) which was shown to lack exon 8 and consists of exons 7-9-10 only (Fig. 2B). These results confirm that the c.309+1G>A splice donor site mutation functionally results in exon 8 skipping (r.193-309del). The resulting mRNA is an in-frame mRNA, but is predicted to lack 39 functionally important amino acids (p.(Asp65_Ile103del)) from the TNNT1 troponin domain (Fig. 2). To identify a second pathogenic*TNNT1* mutation in the proband we performed gene dosage analysis, using quantitative real-time PCR. This revealed a heterozygous*TNNT1* exon 14 deletion. DNA analysis of the proband's mother confirmed that she was carrier for this deletion.

In conclusion, in addition to the previously reported Amish G579T founder mutation in exon 11 (NM_003283.4:c.538G>T p.(Glu189*)), this study identifies two new*TNNT1* NM mutations in three related individuals outside the old order Amish. Most of the clinical features are very similar to ANM, in particular the severe progressive contractures of the extremities, severe pectus carinatum, rigid chest deformities and respiratory insufficiency in the proband. In addition, the proband from our study showed severe kyphosis and diaphragmatic herniation. This latter feature has not been reported before in NM and may reflect thinning and weakness of the diaphragm in the course of the disease. Hypotrophy of the diaphragm has also been observed in*TNNT1* knockout mice (Feng et al. 2009). The cases reported here show an overall slightly milder phenotype than the ANM cases. Trembling of the jaw or lower extremities as reported in the ANM patients were not seen in the Dutch cases. Besides, the ANM patients died at the age of 2, whereas the index case is still alive and the cousins died at the ages of 3 and 5, respectively. These differences may reflect ‘normal’ clinical variability in the condition or, alternatively, may reflect different functional effects of the separate mutations on residual TNNT1 protein function.

The ANM cases were all homozygous for a nonsense mutation in exon 11 of*TNNT1* (Fig. 2C). In these patients, in all likelihood mutated transcripts from both*TNNT1* alleles would be targeted for degradation by nonsense-mediated decay (NMD), resulting in total absence of functional TNNT1 protein and likely a very severe phenotype.

In the cases described in our report, both deceased cousins were homozygous for a*TNNT1* splice site mutation resulting in exon 8 skipping from the*TNNT1* mRNA resulting in shortened in-frame transcripts. These shortened transcripts miss an important part of the functionally important troponin domain. The proband from our study has an exon 14 deletion in addition to the exon 8 skipping mutation. This exon 14 deletion could lead to absence of mutated transcripts through NMD or, alternatively to C terminally shortened transcripts. For both mutations described in our study, we cannot exclude that these shortened transcripts are translated into shortened TNNT1 protein with some residual activity. This possible residual protein activity may explain phenotypic differences between the ANM cases and the ones presented in our study.

Whether clinical differences are real and attributable to genotypic differences is something we feel is too speculative to conclude from these small numbers of patients. More cases need to be reported to further delineate a possible genotype–phenotype correlation.

Our findings show that NM caused by*TNNT1* mutations is not restricted to the Amish population and that the clinically relevant spectrum of*TNNT1* mutations also includes partial gene deletions. The characteristic clinical phenotype with chest deformities and progressive contractures may be important in directing*TNNT1* mutation analysis in NM patients.
